# Assessment of Health Warning Label Compliance on Smokeless Tobacco Products in Bhubaneswar, India

**DOI:** 10.7759/cureus.116986

**Published:** 2026-09-27

**Authors:** Sulagna Pattajoshi, Pratap K Jena, Ipsa Mohapatra

**Affiliations:** 1 Public Health, Kalinga Institute of Industrial Technology (Deemed to be University), Bhubaneswar, IND; 2 Health Care Management, Swiss School of Business and Management Geneva, Geneva, CHE; 3 Community Medicine, Kalinga Institute of Medical Sciences, Bhubaneswar, IND

**Keywords:** bhubaneswar, compliance, cotpa, health warning labels, packaging and labelling, smokeless tobacco, tobacco control

## Abstract

Introduction: Use of smokeless tobacco (SLT) is a serious public health problem in India. Health warning labels (HWLs) play a vital role in imparting information regarding the adverse health effects of tobacco use. Despite the statutory requirement under the Cigarettes and Other Tobacco Products Act (COTPA), there are reports of non-compliance with HWLs in terms of size, position, language, and presentation. The present study aimed to assess compliance with HWLs of SLT products sold by retail shops in Bhubaneswar, India.

Methods: A quantitative observational cross-sectional study was conducted using multistage cluster random sampling in 30 wards of Bhubaneswar Municipal Corporation from August 2025 to July 2026. A total of 515 SLT packs collected from retail outlets were assessed for HWL compliance using an observational checklist. Proportions are reported with 95% confidence intervals (CIs); zone differences were tested using design-based methods (Rao-Scott chi-square test and survey regression) accounting for clustering within wards.

Results: None of the packages assessed fulfilled all HWL requirements, while 496 (96.3%; 95% CI: 93.9-98.0) packages had at least one type of HWL violation. Only 101 (19.6%; 95% CI: 17.2-22.3) packages had a warning at the prescribed top position, while 271 (52.6%; 95% CI: 47.5-57.7) had a warning in the English language, as compared to 14 (2.7%; 95% CI: 1.5-4.5) packages that had a warning in English and Odia languages. Only 5.8% (95% CI: 4.0-8.1) of the packages complied with the requirement of coverage of HWL of ≥85% of the principal display area, while 88.0% of the packages failed to meet the same. Three HWL violations were most frequently observed, occurring in 197 of 515 packs (38.3%; 95% CI: 34.3-42.3), while ≥4 violations occurred in 149 (30.0%; 95% CI: 26.6-33.7) of 496 non-compliant packs. No significant variation was found in compliance across the geographic zones after accounting for clustering within wards (Rao-Scott p = 0.825).

Conclusion: The study found that HWLs of SLT products in Bhubaneswar were largely non-compliant with statutory requirements, with multiple violations in terms of coverage, position, and language. There is a need for strict regulatory and surveillance measures to ensure HWL compliance by SLT manufacturers.

## Introduction

Tobacco use is one of the major causes of morbidity and mortality, and smokeless tobacco (SLT) plays a significant role in India. Khaini, gutkha, zarda, and other localised forms of chewing tobacco are easily accessible at retail shops and are infamous for causing oral cancer, cardiovascular diseases, periodontal disease, and a host of other ailments. Given the wide variety of packaging, product forms, and informal retail outlets, effective packaging regulation assumes significant importance. Health warning labels (HWL) offer a solution by disseminating relevant information about the harmful effects of tobacco consumption directly at the point of sale and consumption. The World Health Organisation's Framework Convention on Tobacco Control endorses prominent, clear, and pictorial HWL covering a large percentage of the tobacco packaging as an effective means to communicate health hazards [1]. Indian legislation comprises the Cigarettes and Other Tobacco Products Act (COTPA) 2003 and the Cigarettes and Other Tobacco Products (Packaging and Labelling) Rules 2008 and subsequent amendments, which govern the sale and distribution of all tobacco products. Section 7 of the Act mandates specific pictorial and textual health warnings on all tobacco packages that are sold, supplied, or distributed in India [2]. Further amendments reinforced pictorial health warnings on all tobacco packages, as well as warnings concerning SLT. The current regulations require warnings to cover at least 85% of the main display surface with regard to the inclusion of pictorial warnings, language, placements, and general instructions for manufacturers [3]. Additionally, the Government of India made several changes to the warnings and their rotations, implying the continuous need for monitoring products in the market [3].

While India's legislative framework mandates HWL on all tobacco packaging, adherence at the retail level remains poor. An analysis of SLT and bidi packages across five Indian states found that, of 133 surveyed SLT products, only 1% met all the indicators assessed, including warning location, warning elements, and warning size [4]. Incomplete warnings, non-uniform packaging, unclear print, and expired warning labels were among the issues identified. In Mangaluru, SLT packages carried warnings covering only 70.6% of the required surface area on average, and half of the sampled products did not follow the prescribed warning rotation [5]. A study in Udupi similarly found that only 8% of surveyed tobacco packages complied with the warning-size requirements under Section 7, with pictorial warnings often unclear or split [6]. In Delhi, some SLT packages carried no pictorial warning at all, while others displayed promotional content instead [7], and a later assessment following the 2020 Third Amendment Rules found that SLT and foreign-origin products in the city remained poorly compliant, particularly for manufacturing-date and country-of-origin disclosures [8]. More recently, a 2025 survey in the pilgrimage town of Deoghar, Jharkhand, found that 92.8% of SLT outlets sold packs with no pictorial warning printed at all [9], and a regional pack analysis across Bangladesh, India, and Pakistan found that 93% of Indian SLT packs lacked adequate health warnings, meeting the criteria for illicit packaging under national law [10]. Such inadequacies are not merely a matter of legal non-compliance: warnings that are missing, misplaced, or hard to see are also less likely to be noticed by users. Studies among Indian SLT users have identified this as a key reason warnings go unnoticed or are ignored [11,12], and increased warning size together with more consistent rotation has been shown to improve warning efficacy [12].

Studies from multiple Indian states point to significant and recurring HWL violations, suggesting that similar patterns are likely elsewhere in the country, yet a recent, city-wide survey of SLT-specific HWL compliance in Odisha has been lacking. An earlier Bhubaneswar study examined tobacco warnings around educational institutions and documented incomplete, distorted, faded, and out-of-rotation warnings [13], but it was confined to vendors within a 100-yard radius of schools and covered both SLT and smoking products together.

The current study focuses on examining HWL across various retail outlets within the capital city of Odisha to detect warning inadequacy and identify possible regional and vendor-specific differences. By classifying the warning labels into levels of compliance, this study goes beyond simply noting violations and aims to identify specific factors that contribute to them. It also aims to highlight areas that may require specific policy interventions and increased surveillance in order to improve warning efficacy. The study seeks to add to the growing literature on Indian tobacco packaging and HWL, with possible implications for the enforcement of COTPA, improved compliance with warning labelling among manufacturers, and more consumer awareness in general regarding the health consequences of SLT.

The study aims to evaluate the degree of compliance with HWL regulations by SLT sold through retail outlets in Bhubaneswar city with respect to the COTPA and the pertinent rules regarding tobacco packaging. It will classify SLT packages according to the level of HWL compliance with respect to the statutory requirements. Finally, it aims to quantify the frequency and nature of HWL violations in SLT at sale, as well as determine differences in patterns of violations between different geographical locations.

## Materials and methods

This study used a quantitative, observational, cross-sectional design to assess HWL compliance for SLT products in retail outlets in Bhubaneswar city. The study was conducted in Bhubaneswar Municipal Corporation (BMC), which had 67 municipal wards spread across the north, southeast, and southwest zones. The study included paan shops, kiosks, grocery/general stores, convenience stores, mixed outlets, tea stalls/small eateries, and supermarkets. The study was conducted over a period of one year, from August 2025 to July 2026, which included the time for preparatory activities, developing and pilot testing the observational tool, collecting data (samples and observations) in the field, and entering, cleaning, and analysing the data. The packs were continuously collected throughout the study period and assessed as per availability. The study population consisted of retail outlets selling SLT products in the municipal area of Bhubaneswar. The primary units of sampling were retail outlets, and the collected SLT products, including khaini, gutkha, pan masala with tobacco, Zarda, chewing tobacco, and other SLT products, were assessed for HWL compliance. Non-tobacco products with similar packaging or branding to tobacco products were also observed when relevant. Using the expected prevalence of HWL non-compliance of 73% [11], a 95% confidence level, and 5% absolute precision, the sample size was calculated using the formula:

*n=(z2p(1-p))/E2).*

This gave the value of 303. A design effect of 1.5 was applied to account for cluster sampling, giving a sample size of 454.5 (303 × 1.5), which was rounded up to 455. An additional 10% was added to account for potential non-response and inaccessible outlets, resulting in a calculated sample size of 500.5 (455 + 45.5), which was rounded up to 501. A final sample size of 515 retail outlets was ultimately included in the study. A multistage cluster random sampling procedure was used to select the sample. The study sites were stratified according to the three zones of the BMC, and wards were randomly selected from each zone (north: 8; southeast: 11; southwest: 11; total 30 wards), followed by the random selection of retail outlets (171, 172, and 172, respectively) using the walking protocol.

The study included retail outlets selling at least one type of SLT product, including paan shops, kiosks, grocery/general stores, convenience stores, tea stalls/small eateries, and supermarkets. The study excluded outlets that did not sell SLT products, warehouses that did not sell directly to consumers, permanently closed outlets, outlets that were closed on two separate visits, and outlets that declined to be observed after explaining the purpose of the survey.

The selected wards and retail outlet clusters were identified and visited by the field investigators using the walking protocol procedure, followed in the Tobacco Pack Surveillance System (TPackSS, Institute for Global Tobacco Control, Johns Hopkins Bloomberg School of Public Health, Baltimore, MD) [4]. The field investigators identified the eligible retail outlets, observed the SLT products on display, and collected samples considering differences in size, brand name, and product type while avoiding duplication of packs and ensuring that at least one pack was collected from each selected retail outlet. The investigators assessed the HWLs of the collected products in terms of coverage, position, language, visibility, and printing characteristics. Wherever possible, photographs of the pack displays were taken while ensuring that no personal or other identifying information was captured. The data were collected using the developed observational checklist. The observational checklist was developed based on the COTPA, the relevant Packaging and Labelling Rules, the recommendations of the WHO Framework Convention on Tobacco Control, the TPackSs, and published literature. The observational checklist had sections on retail outlet information, SLT product information, HWL assessment, and observational notes.

Prior to the commencement of the fieldwork, the observational checklist was reviewed by experts in the field of community medicine, public health, epidemiology, and tobacco control. The recommendations of the experts were considered before the commencement of the fieldwork.

The study obtained ethical approval from the Institutional Ethics Committee of KIMS (Ref. No. KIIT/KIMS/IEC/2634/2026) before the commencement of the study. The study involved observing the retail outlets and the SLT products. The study did not collect any personal information from the customers. Photographs were taken of the retail outlets and the tobacco products with permission, ensuring that no personal information was captured. The collected information and the photographs were kept confidential and used for the purposes of the study only. The study followed the ethical guidelines of the KIMS ethical committee and the Indian Council of Medical Research (ICMR).

A pilot study was conducted in 25 retail outlets that were not included in the study clusters to determine the feasibility of the study, clarify the instructions, review the consistency of observer assessments, and determine the time taken to complete the observational checklist. Double-observer ratings were not recorded during the pilot or the main survey; hence, inter-observer agreement could not be quantified.

The data were collected by the field investigators who visited the selected retail outlets. The investigators assessed the collected SLT products using the developed observational checklist. The information collected included the outlet's type, location, and the observed SLT products and their HWLs. Each retail outlet visit lasted 10-15 minutes. The primary outcome of the study was the overall HWL compliance of the observed SLT products. The study also determined the proportion of packs complying with the warning coverage, position, language, clarity, and visibility, and the patterns of HWL non-compliance. The study also assessed HWL compliance across the geographical zones. The completed observational checklists were reviewed on a daily basis, and the data were checked for completeness, consistency, range checks, duplicate records, and missing values.

The study variables included overall compliance, size compliance, position compliance, language compliance, clarity compliance, and visibility compliance. The completed observational checklists were reviewed on a daily basis to determine their level of completeness and consistency. The data were entered into Microsoft Excel (Microsoft® Corp., Redmond, WA) spreadsheets and analysed using R software (version 4.6.1; R Foundation for Statistical Computing, Vienna, Austria) with the survey (version 4.5) and lme4 (version 2.0.6) packages. The descriptive statistics of the data were generated, including the frequencies of categorical variables and the means and standard deviations or medians and interquartile ranges for continuous variables. Proportions of HWL compliance were estimated with 95% confidence intervals (CIs). To account for the multistage cluster sampling design, inferential analyses specified the three BMC zones as strata and wards as primary sampling units (clusters); packs collected from the same outlet were nested within wards. Wards were assigned to zones according to the official BMC ward-zone classification. Because outlet sampling frames for individual wards were unavailable, the analysis was unweighted. CIs for proportions were estimated using the Korn-Graubard method, which incorporates the design effect, and the exact Clopper-Pearson method was used where the observed proportion was 0%. The design effect (DEFF) and ward-level intra-cluster correlation coefficient (ICC) were estimated to quantify clustering. The association between HWL compliance category (partial versus poor) and geographic zone was assessed using the Rao-Scott design-adjusted chi-square test, and the mean number of HWL violations across zones was compared using design-based linear regression with a Wald F-test. As sensitivity analyses, mixed-effects logistic and linear regression models with a random intercept for ward were fitted, and zone effects were assessed using likelihood-ratio tests. Statistical significance was set at p < 0.05.

## Results

Overall HWL compliance

A total of 515 SLT product packages from the surveyed retail outlets were analysed to assess compliance with the HWL requirements. Out of 515 packs, 496 were analysed, while 19 were excluded from analysis. Overall compliance was evaluated by checking if the warning characteristics were present and correctly placed, worded, sized, and printed. None of the investigated packages met all HWL requirements (0.0%; 95% CI: 0.0-0.7). In total, 496 (96.3%; 95% CI: 93.9-98.0) packages were non-compliant, and 19 (3.7%) packages were non-assessable packs (Table 1). Specifically, 347 (70.0%) of the non-compliant packages had 1-3 violated warning features, indicating a partially/moderately non-compliant status, while 149 (30.0%; 95% CI: 26.6-33.7) had ≥4 violated warning features, indicating a poorly non-compliant status (Figure 1).

**Table 1:** Classification of collected sample packs according to overall health warning label compliance (N = 515) *Percentage calculated among the 496 packs for which overall health warning label (HWL) compliance could be assessed. Note: Although the operational category of partial/moderate non-compliance was defined as 1-3 violations, the observed dataset contained packs with 2 or 3 violations in this group; no assessable pack had 0 or 1 violation due to co-occurrence of violations.

Level of Classification	HWL Compliance Category	Operational Definition	Frequency (n)	Percentage (%)
Primary classification	Compliant	0 HWL violations	0	0
	Non-compliant	≥1 HWL violation	496	96.3
	Not applicable	Non-tobacco products	19	3.7
Further classification of non-compliant packs (n = 496)	Partially/Moderately non-compliant	1-3 HWL violations	347	70.0*
	Poorly non-compliant	≥4 HWL violations	149	30.0*

**Figure 1:**
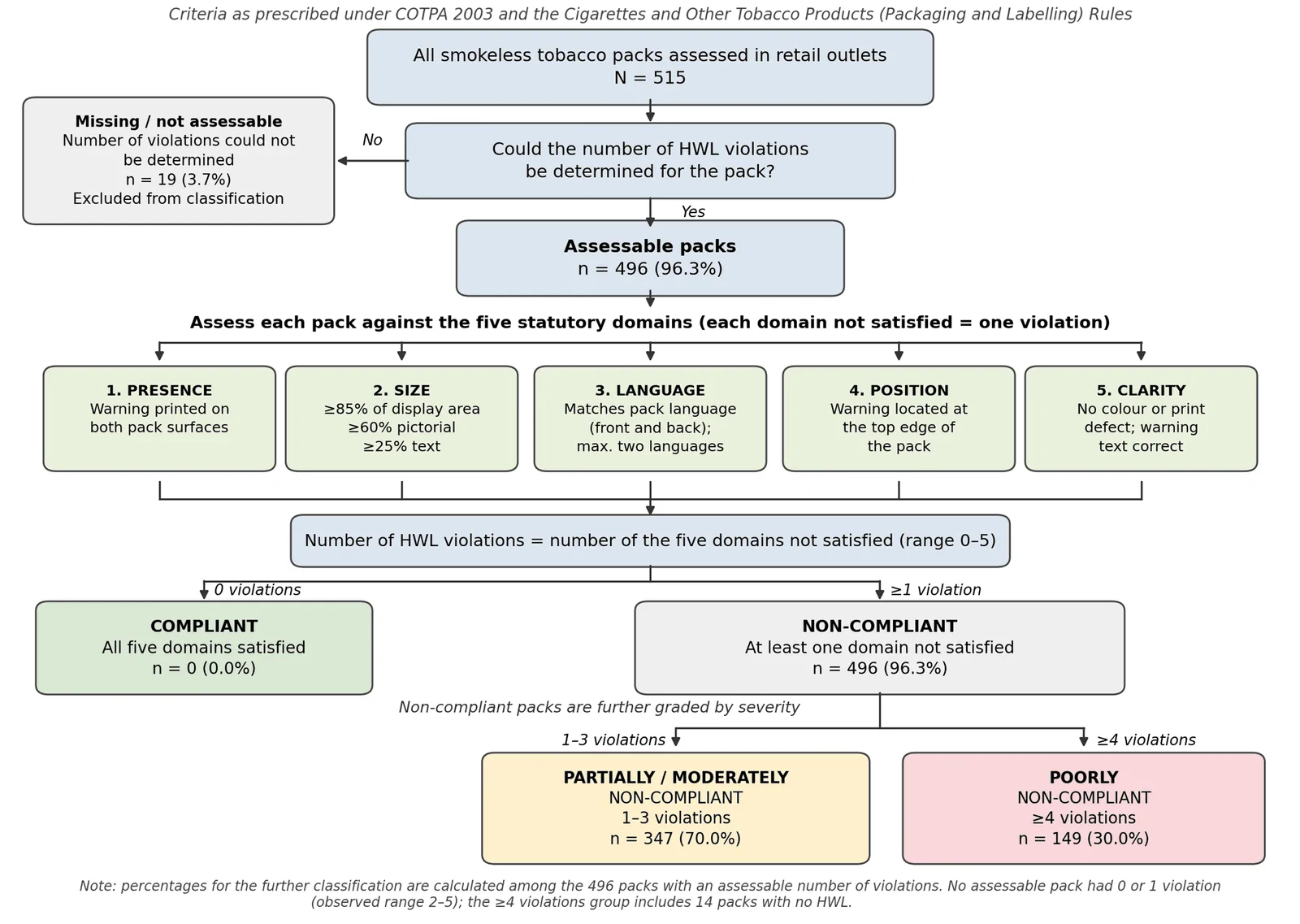
Flowchart showing the levels of health warning label (HWL) compliance The criteria are as per the COTPA Act 2003 [2]. No pack had 0 or 1 violation. The assessable packs are 496, excluding 19 packs that are non-tobacco.

Position of HWLs

The COTPA-prescribed position of HWL (top) was found in only 101 (19.6%; 95% CI: 17.2-22.3) packages. The warning was at the bottom of the package in 129 (25%) and in the middle in 129 (25%) packages. HWL was split in 30 (5.8%) packages and absent in 14 (2.7%) of the packages. A total of 112 (21.7%) packages were not assessable for the position of HWL (Figure 2). Overall, less than a fifth of the HWLs that were tested appeared in the prescribed position on the packs, suggesting that the industry does not follow the law in relation to HWL positioning.

**Figure 2:**
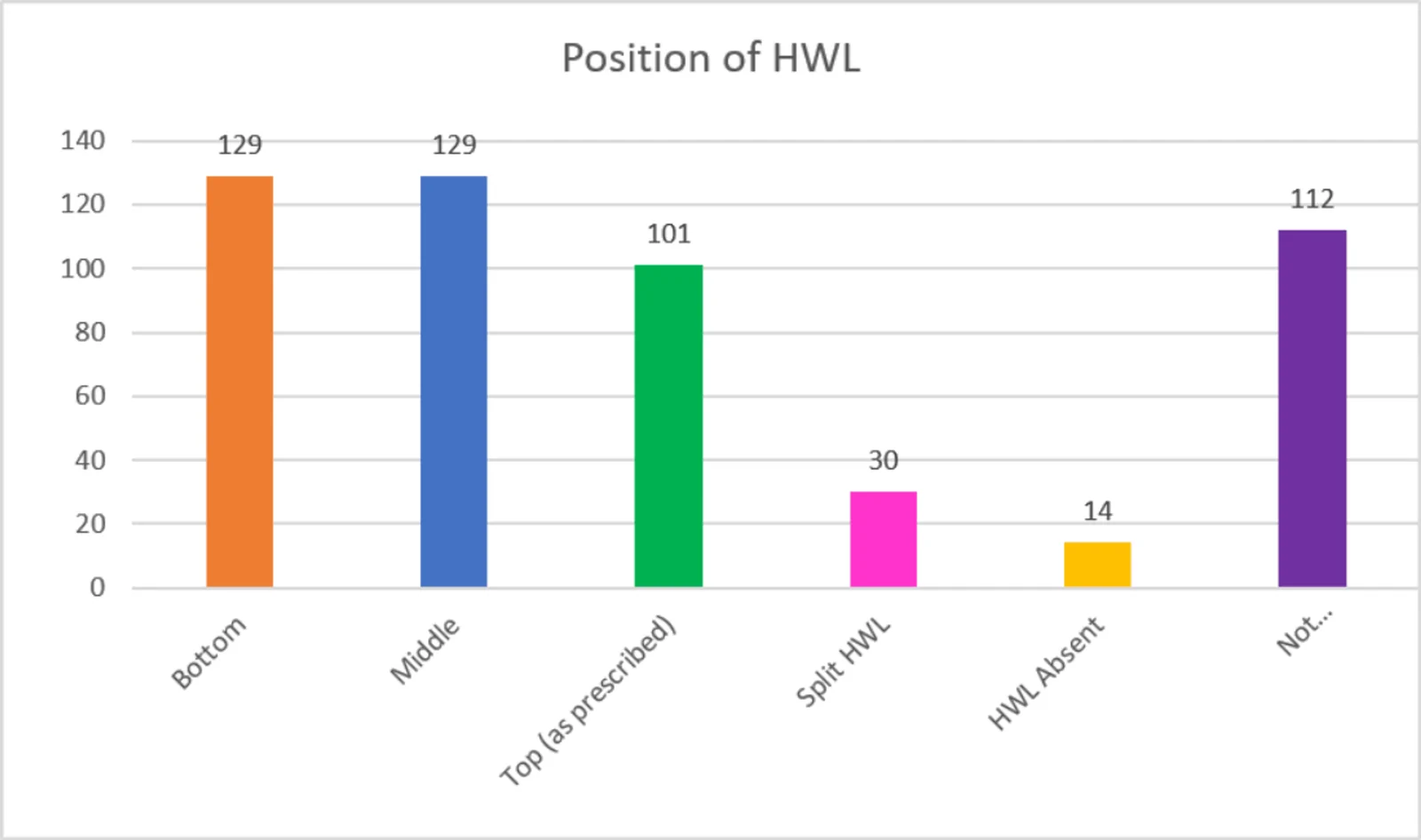
Position of the health warning label on the principal display area (N = 515)

Language of HWLs

English was the most prevalent language used on the packages, as reported in 271 (52.6%; 95% CI: 47.5-57.7) packages, followed by Hindi-only warnings that appeared on 69 (13.4%) packages and English-Hindi bilingual warnings that appeared on 35 (6.8%) packages. Furthermore, only 14 (2.7%; 95% CI: 1.5-4.5) packages had an English and Odia language combination. The Indian packaging and labelling requirements specify the use of English, Hindi, and appropriate regional languages, and the language combination should correspond to the language used on the package. For 126 (24.5%) packages, the language on the packages was either missing or unassessable (Table 2). The low representation of Odia language was unexpected given the study's regional context, which implied that there was limited use of regional language in HWL.

**Table 2:** Language used for the health warning label (HWL) (N = 515) *Non-tobacco packs and missing HWL

Language of HWL	Frequency n (%)
English	271 (52.6)
Hindi	69 (13.4)
English + Hindi	35 (6.8)
English + Odia	14 (2.7)
Not assessable*	126 (24.5)
Total	515 (100)

HWL coverage and printing clarity and other HWL deficiencies

Compliance with the requirement that HWL should cover at least 85% of the area of the pack was particularly poor. Only 30 (5.8%; 95% CI: 4.0-8.1) packages complied with the 85% guideline, while 453 (88.0%; 95% CI: 85.0-90.5) did not. Another 32 (6.4%) could not be assessed due to the inability to quantify the proportion of the principal display area (Table 3). In other words, HWL coverage was amongst the most frequent deficiencies detected. Printing problems were frequent, with the most common being HWL obscured by brand logos, detected in 96 (18.6%; 95% CI: 15.7-22.0) packages. This was followed by HWL cropped at the warning area in 81 (15.7%; 95%CI: 12.4-19.8) packages and split warnings and missing quit-line in 67 (13.0%; 95%CI: 9.3-17.9) packages each. Poor contrast or low tint was found in 39 (7.6%; 95%CI: 5.6-10.3) of the packages, while 119 (23.1%; 95%CI: 20.4-26.2) had no printing deficiencies detected (Table 3). Therefore, it was evident that, besides the size and location, there were other issues associated with HWL.

**Table 3:** Health warning label (HWL) coverage and printing clarity and other HWL deficiencies (N = 515) * Non-tobacco packs and missing HWL; # Multiple response variable. Multiple printing inaccuracies could be recorded for a single pack; therefore, percentages do not sum to 100%.

Health Warning Label Covers ≥85% of the Principal Display Area	Frequency n (%)
Yes	30 (5.8)
No	452 (87.8)
Not assessable	33 (6.4)
Total	515 (100)
Printing inaccuracy#	Frequency n (%)
Obstruction due to branding	103 (20.0)
Cropping issues	65 (12.6)
Not assessable*	81 (15.7)
Split health warning label	67 (13.0)
Missing text/Quitline	67 (13.0)
Low tint /poor print quality	49 (9.5)
No printing inaccuracy observed	179 (34.8)

Geographic variation in HWL compliance

Among the 496 assessable packages, 347 (70.0%) were partially/moderately compliant, and 149 (30.0%) were poorly compliant. Poor compliance was observed in 29.3% (95% CI: 18.3-42.5) of packs from the north zone, 29.5% (95% CI: 24.3-35.2) from the southeast zone, and 31.6% (95% CI: 26.7-36.8) from the southwest zone. After accounting for clustering of packs within wards, the difference between geographic zones was not statistically significant (Rao-Scott F = 0.18, p = 0.825, Table 5). The mean number of HWL violations was 3.14 ± 0.85 in the north zone, 3.04 ± 0.90 in the southeast zone, and 3.07 ± 0.88 in the southwest zone; this difference was also not statistically significant (design-based regression, F = 0.42, p = 0.660, Table 4). Clustering of outcomes within wards was negligible (ward-level ICC ≤ 0.06; design effects: 0.49-1.25), and mixed-effects models with a random intercept for ward gave consistent results (likelihood-ratio test p = 0.903 for compliance category and p = 0.731 for number of violations).

**Table 4:** Association between health warning label (HWL) compliance and geographic zone (Rao-Scott design-adjusted chi-square test) (N = 496) *Rao-Scott design-adjusted chi-square test with zones as strata and wards as clusters (30 wards; design degrees of freedom = 27). Zones were assigned according to the official Bhubaneswar Municipal Corporation ward-zone classification.

Geographic Zone	Partially Compliant n (%)	Poor Compliance n (%)	Total	Rao-Scott F*	p-value
North zone	41 (70.7)	17 (29.3)	58	0.18	0.825
Southeast zone	215 (70.5)	90 (29.5)	305		
Southwest zone	91 (68.4)	42 (31.6)	133		
Total	347 (70.0)	149 (30.0)	496		

**Table 5:** Comparative analysis of health warning label violations across geographic zones (N = 496) *Design-based linear regression (Wald F-test) with zones as strata and wards as clusters.

Geographic Zone	Mean No. of HWL Violations ± SD	Median (IQR)	Minimum	Maximum	p-value*
North zone	3.14 ± 0.85	3 (3-4)	2	5	
Southeast zone	3.04 ± 0.90	3 (2-4)	2	5	
Southwest zone	3.07 ± 0.88	3 (2-4)	2	5	
Overall	3.06 ± 0.88	3 (2-4)	2	5	0.660

Identification of factors for poor HWL compliance

In the multivariable design-based model, product category was independently associated with poor compliance (overall p < 0.001). Compared with pan masala, mawa packs had nearly fourfold higher odds of poor compliance (AOR: 3.90, 95% CI: 1.72-8.83), and gutka packs showed a similar trend that did not reach statistical significance (AOR: 2.44, 95% CI: 0.97-6.12). Retail outlet type and geographic zone were not significantly associated with poor compliance in the multivariable model (Table 6).

**Table 6:** Factors associated with poor HWL compliance (≥4 violations) among assessable packs (N = 496) Survey logistic regression with zones as strata and 30 wards as clusters (unweighted). p-values on shaded rows are overall design-based Wald F-tests for the factor; p-values on category rows compare with the reference category. Permanent kiosks (n=4) were combined with mobile/temporary vendors. OR, odds ratio; AOR, adjusted odds ratio (all factors in the table entered simultaneously); CI, confidence interval

Factor/category	n	Poor, n (%)	Crude OR (95% CI)	p	AOR (95% CI)	p
Product category				<0.001		<0.001
Pan Masala	164	44 (26.8)	1.00 (ref)		1.00 (ref)	
Khaini	131	45 (34.4)	1.43 (0.87-2.35)	0.154	1.07 (0.65-1.75)	0.773
Tobacco	77	16 (20.8)	0.72 (0.33-1.55)	0.378	0.73 (0.30-1.76)	0.45
Zarda Patti	72	17 (23.6)	0.84 (0.41-1.75)	0.631	0.87 (0.39-1.93)	0.71
Mawa	37	20 (54.1)	3.21 (1.66-6.20)	0.001	3.90 (1.72-8.83)	0.003
Gutka	15	7 (46.7)	2.39 (0.99-5.74)	0.052	2.44 (0.97-6.12)	0.057
Pack text includes Odia				0.036		0.058
No	411	112 (27.3)	1.00 (ref)		1.00 (ref)	
Yes	85	37 (43.5)	2.06 (1.05-4.02)	0.036	2.03 (0.97-4.24)	0.058
Product name printed				0.054		0.079
Yes	450	141 (31.3)	1.00 (ref)		1.00 (ref)	
No	46	8 (17.4)	0.46 (0.21-1.01)	0.054	0.42 (0.16-1.13)	0.079
Date of manufacture printed				0.091		0.522
Yes	97	24 (24.7)	1.00 (ref)		1.00 (ref)	
No	399	125 (31.3)	1.39 (0.94-2.04)	0.091	1.13 (0.75-1.72)	0.522
Retail outlet type				0.61		0.867
Paan/betel shop	231	65 (28.1)	1.00 (ref)		1.00 (ref)	
Tea stall/small eatery	162	49 (30.2)	1.11 (0.80-1.54)	0.528	1.08 (0.78-1.49)	0.628
Grocery/general store	76	27 (35.5)	1.41 (0.75-2.64)	0.274	1.35 (0.61-3.01)	0.431
Kiosk/mobile vendor	27	8 (29.6)	1.08 (0.55-2.08)	0.822	0.98 (0.48-2.01)	0.951
Geographic zone				0.792		0.873
Southeast	305	90 (29.5)	1.00 (ref)		1.00 (ref)	
North	58	17 (29.3)	0.99 (0.57-1.71)	0.972	0.95 (0.53-1.72)	0.86
Southwest	133	42 (31.6)	1.10 (0.80-1.51)	0.529	1.06 (0.76-1.48)	0.724

Sensitivity and secondary analysis

In the secondary analysis of the number of violations (Table 7), product category (p = 0.004) was associated with the number of violations, whereas outlet type and zone were not. There was no evidence of collinearity among the factors (all GVIF^(1/(2·df)) ≤1.32), and the ward random-effect variance in the mixed model was effectively zero, consistent with the negligible clustering observed in the main analysis.

**Table 7:** Sensitivity and secondary analyses (N = 496) Mixed-model AOR: multivariable logistic regression with a random intercept for ward; LRT, likelihood-ratio test for the factor. Adjusted mean difference: design-based linear regression of the number of HWL violations on all factors (zones as strata, wards as clusters); p-values on shaded rows are overall Wald F-tests.

Factor/category	Mixed-model AOR (95% CI)	LRT p	Adjusted mean difference in violations (95% CI)	p
Product category		0.002		0.004
Pan Masala	1.00 (ref)		0 (ref)	
Khaini	1.07 (0.55-2.07)		-0.03 (-0.21 to 0.16)	0.778
Tobacco	0.73 (0.37-1.45)		0.11 (-0.16 to 0.37)	0.399
Zarda Patti	0.87 (0.45-1.68)		0.32 (0.10 to 0.54)	0.009
Mawa	3.90 (1.75-8.69)		0.45 (0.09 to 0.80)	0.017
Gutka	2.44 (0.81-7.31)		0.03 (-0.38 to 0.45)	0.863
Retail outlet type		0.796		0.612
Paan/betel shop	1.00 (ref)		0 (ref)	
Tea stall/small eatery	1.08 (0.68-1.71)		-0.06 (-0.21 to 0.08)	0.369
Grocery/general store	1.35 (0.74-2.46)		0.11 (-0.16 to 0.38)	0.397
Kiosk/mobile vendor	0.98 (0.37-2.60)		-0.02 (-0.21 to 0.16)	0.790
Geographic zone		0.956		0.866
Southeast	1.00 (ref)		0 (ref)	
North	0.95 (0.48-1.88)		0.05 (-0.16 to 0.27)	0.599
Southwest	1.06 (0.65-1.72)		0.01 (-0.17 to 0.19)	0.888

## Discussion

The current study highlights the non-compliance with statutory HWL requirements as seen among the collected SLT products sold at the selected retail outlets in Bhubaneswar, with none of the evaluated packages meeting the requirements. The study indicates that 96.3% of the assessed packages had at least one violation, while 70.0% of non-compliant packs had one to three violations, and 30% had at least four violations, suggesting multiple violations rather than isolated ones. The current findings support the general tobacco control literature surrounding the need for prominent, highly visible, and noticeable health warnings on all tobacco packaging as a way of communicating the various health risks associated with tobacco use [1]. Additionally, the current findings indicate major gaps in HWL implementation, even though the legal requirements by COTPA 2003 and subsequent cigarette and package labelling guidelines had been put in place [2,3]. Notably, the location of the warning was often incorrect, with only 19.6% of packages having warnings at the top of the package as required. Meanwhile, 25% had warnings at the bottom, and 25% had warnings in the middle, with 5.8% having split warnings and 2.7% having no warning at all. Similarly, previous Indian HWL studies had also indicated significant variation in warning location, with most packages having warnings at the bottom [5-7]. In fact, the current study concurs with the Karnataka state findings regarding violations of warning location, size, and presentation among various tobacco packages [5-7]. The relatively low percentage of correct warning location recorded in the current study reduces the prominence of health warnings and their potential preventive impact.

Additionally, the HWL coverage showed an even greater level of non-compliance, with only 5.8% of the assessed packages having warnings covering at least 85% of the package. Meanwhile, 88.0% of the packages had warnings with coverage below the required threshold, an issue which had also been documented in other Indian HWL studies. For instance, the five-state assessment of SLT products and bidis indicates widespread violation of warning size and presentation requirements [5], while the Mangaluru study also reports similar issues with HWL size and presentation [6]. Notably, the issue of coverage is a key concern since smaller warning size may lead to the diminished effect of the warning compared to larger warning sizes. Additionally, language posed another significant challenge, with most of the warnings being in English (52.6%), while only 2.7% of the packages had both English and Odia language printed on them. The issue of language is pertinent since health warnings are supposed to be understood by the majority of the population and not in a language only a minority can comprehend. In fact, the current findings concur with the general Indian HWL literature, indicating wide variation in warning presentation and highlighting the need for warnings to be noticeable and understandable [7-10]. Therefore, the current study's findings on the low percentage of packages with warnings in Odia highlight the limitation of language in health warning dissemination.

The current study also highlights major printing and presentation issues, with most packages having warnings either cropped, obstructed, or split. The current findings are similar to previous Indian HWL studies, which had also recorded high levels of warnings' obstruction, distortion, cropping, and overall poor presentation [5,7,8]. For instance, the Delhi study on HWL compliance indicated major violations of the warnings' presentation and general pack labelling requirements [8]. Indeed, the current study highlights several concerns with respect to HWL among local SLT packages. The main concern is that, even with the existence of legal HWL requirements, the tobacco industry continues to print warnings in a manner that defeats their purpose. For instance, warnings can be easily ignored, obstructed, and difficult to read due to poor presentation. The current study builds upon the previous local study on HWL among various tobacco packages around educational institutions by highlighting similar issues recorded in the wider marketplace rather than one specific location. In fact, a previous HWL study among local college smokers also found similar issues with obstructed, incomplete, distorted, split, and out-of-rotation warnings [11]. Therefore, it can be argued that, while the local study focused on college students and one specific location, the current study provides more generalizable findings on issues with HWL within Bhubaneswar. Notably, the current study did not find significant geographical differences in HWL compliance across the different zones, with most packages having violations. However, HWL non-compliance was higher in the southwestern zone than the northern zone, though the difference was not statistically significant (Rao-Scott p = 0.825). This implies that the issue of poor HWL compliance affects the whole of Bhubaneswar, with the differences recorded among the different zones being minimal. As a result, the current study provides empirical justification for the need to enforce greater monitoring and surveillance of HWL within the city. The findings are consistent with cluster-based compliance studies conducted at points-of-sale and retail outlets in settings of Ethiopia and China where multi-stage cluster sampling techniques were employed. The regression models were applied accounting for the clustered design to identify factors associated with non-compliance and other retail-level outcomes, reinforcing the appropriateness of cluster-adjusted regression approaches in point-of-sale compliance research [13,14]. Overall, the current study indicates the need for more extensive and frequent monitoring of HWL compliance among local SLT products to ensure the tobacco industry follows the regulations around HWL as intended by the law [2-4,10].

The study had several limitations that should be considered while interpreting its findings. Its cross-sectional design provided a snapshot of HWL compliance during the study period and therefore could not assess changes in compliance over time or determine whether regulatory amendments subsequently influenced packaging practices. The assessment was restricted to retail outlets within BMC and, therefore, the findings may not be generalizable to rural areas, other cities of Odisha, or other states with different retail and enforcement environments. The study assessed products available at selected retail outlets and may not have captured SLT products sold through informal, wholesale, online, or other distribution channels. Some packages were also classified as not assessable because of the presence of non-tobacco products in the sample. Assessment of characteristics such as clarity and printing quality involved structured observational judgement and may have been subject to some degree of observer variation, although expert review, pilot testing, and trained data collectors were used to improve consistency. However, double-observer ratings were not recorded during the pilot or the main survey, so inter-observer agreement (e.g., Cohen's kappa) could not be quantified and some misclassification of judgement-based items cannot be excluded. In addition, because outlet sampling frames for individual wards were unavailable, the analysis was unweighted; although clustering within wards was accounted for in all inferential analyses, the estimates may not fully reflect differences in outlet density between wards. The number of sampled wards also differed across zones, and the north zone contributed fewer packs, resulting in less precise estimates for that zone.

## Conclusions

The study established the extent of non-compliance of collected SLT packages with HWL regulations at the study location. In general, no collected package was compliant with all HWL regulations, and most of them (96.3%) had at least one HWL violation. Notably, the majority of the collected packages had warnings in an incorrect position or provided coverage of the warning area smaller than the stipulated 85%. Furthermore, violations related to HWL language and print quality were observed, with only 14 (2.7%) packages displaying HWLs in both English and Odia. There was also a high prevalence of multiple violations, suggesting that HWL non-compliance in the study location is a serious concern. There was no significant difference found in HWL compliance across the three BMC zones. The current study recommends enhancing monitoring and evaluation activities to ensure that manufacturers meet all HWL requirements so that the intended purpose of health warnings to create awareness of the harms associated with SLT use is served.
